# Quantifying shedding of synthetic fibers from textiles; a source of microplastics released into the environment

**DOI:** 10.1007/s11356-017-0528-7

**Published:** 2017-10-28

**Authors:** Bethanie M. Carney Almroth, Linn Åström, Sofia Roslund, Hanna Petersson, Mats Johansson, Nils-Krister Persson

**Affiliations:** 1 Department of Biological and Environmental Sciences, University of Gothenburg, Box 463, 40503 Göteborg, Sweden; 2 The Swedish School of Textiles, Smart Textiles, University of Borås, SE-501 90 Borås, Sweden

**Keywords:** Microplastics, Fibers, Fabric, Shedding, Synthetic textiles

## Abstract

Microplastics in the environment are a subject of intense research as they pose a potential threat to marine organisms. Plastic fibers from textiles have been indicated as a major source of this type of contaminant, entering the oceans via wastewater and diverse non-point sources. Their presence is also documented in terrestrial samples. In this study, the amount of microfibers shedding from synthetic textiles was measured for three materials (acrylic, nylon, polyester), knit using different gauges and techniques. All textiles were found to shed, but polyester fleece fabrics shed the greatest amounts, averaging 7360 fibers/m^−2^/L^−1^ in one wash, compared with polyester fabrics which shed 87 fibers/m^−2^/L^−1^. We found that loose textile constructions shed more, as did worn fabrics, and high twist yarns are to be preferred for shed reduction. Since fiber from clothing is a potentially important source of microplastics, we suggest that smarter textile construction, prewashing and vacuum exhaustion at production sites, and use of more efficient filters in household washing machines could help mitigate this problem.

## Introduction

Plastic production has increased rapidly since production began in the 1940s and current production in excess of 311 million tons (Org. 2015). Though plastics are essential to modern society, the long-term consequences of such intensive use is largely unknown. Microplastics, sources, fate, and effects, have received increasing attention both from the scientific community as well as from the general public and policy makers. The term “microplastic” normally refers to plastic particles that are < 5 mm in length (Thompson et al. 2004), though the term has more recently been applied to particles that are < 1 mm in size (Browne et al. 2007). Numerous studies demonstrate the ubiquitous presence of microplastics in various compartments of the environment including in water, sediment, terrestrial soil, and biota (Andrady 2011; Browne et al. 2011a, b; Cole et al. 2011; Collignon et al. 2012; Duis and Coors 2016; Enders et al. 2015; Lusher et al. 2013). These microplastics are thought to be hazardous to marine organisms (Della Torre et al. 2014; Lee et al. 2013; Lu et al. 2016; Rochman et al. 2013; Rochman et al. 2014; Wright et al. 2013). Evidence indicates that macroplastics, objects larger than 5 mm in size which typically include plastic litter, will weather and fragment in the environment to form microplastic particles (Lambert and Wagner 2016; Weinstein et al. 2016), but microplastics are also known to enter directly into the environment from multiple sources and routes, including road run off, waste water treatment plants (sewage), and potentially artificial turf (Browne et al. 2011a; Magnusson et al. 2016).

One commonly identified type of microplastic found in environmental samples is fibers, which are thought to originate from textiles. Both natural textile fibers (wool, linen, and cotton) and synthetic textile fibers (polyester, polyamide) are found in the marine environment (Mathalon and Hill 2014; Remy et al. 2015). These synthetic microfibers have also been shown to have negative effects in organisms such as *Daphnia magna* which were found to ingest polyester fibers, with increased mortality as a consequence (Jemec et al. 2016). A recent publication indicated that the number of fibers released from washing 6 kg of laundry could reach more than 700,000 fibers (Napper and Thompson 2016). A previous publication reported lower levels in wash effluent; outgoing water from washing machines could contain 100–300 fibers per liter (Browne et al. 2011a), though the information presented in that article is not detailed enough to fully quantify the amount of fibers released from the textiles. In addition, this study did not make use of detergent in washing procedures, for technical reasons, and may therefore present underestimated values.

Washing effluent will in many countries ultimately reach sewage treatment plants. Studies addressing the release of microplastics in sewage effluent indicate that 70–99% of microplastic particles can be retained in sludge, and that technologies at the sewage treatment plant, i.e., sand filters or membrane bioreactors (Magnusson and Norén 2014; Talvitie et al. 2017), can be of importance. However, microplastic fibers are still found in the outgoing effluent and have been reported at levels above 1770 particles per hour (Magnusson and Norén 2014; Magnusson and Wahlberg 2014) (or approximately 0.009 particles/L). These fibers are known to reach aquatic systems directly (Dris et al. 2015; Leslie et al. 2013) as well as terrestrial systems via the spreading of sludge (Habib et al. 1998; Murphy et al. 2016; Zubris and Richards 2005).

The release of synthetic fibers into the environment is not innocuous. There is widespread contamination in environmental habitats and in wildlife. Laboratory evidence (Rochman et al. 2013) suggests that there may be environmental impacts and consequences to wildlife from microplastics (Browne et al. 2015; GESAMP 2015; Rochman et al. 2016), though many of the published studies addressing effects of microplastics focus on “round” microplastics and not fibers, which could potentially have different, shape-dependent effects. Fibers have been found not only in sewage effluent, but have been found to be present in coastal waters and aquatic and marine organisms (De Witte et al. 2014; Desforges et al. 2015; Lusher et al. 2013; Mathalon and Hill 2014; Sussarellu et al. 2016; Wright et al. 2013) as well as terrestrial environments and organisms (Habib et al. 1998; Huerta Lwanga et al. 2016; Palmqvist et al. 2016; Zubris and Richards 2005). Microplastic fibers are also found in products meant for human consumption. This includes blue mussels (De Witte et al. 2014; Rochman et al. 2015), honey (Liebezeit and Liebezeit 2013), table salt (Yang et al. 2015), and beer (Liebezeit and Liebezeit 2014). However, it is important to note that these references provide differing levels of verification in their identification of the described particles, ranging from visual identification to μFTIR analyses.

Since we presume that textiles are important sources of synthetic fibers, based on our knowledge of quantities of fibers in the environment and pathways via which they may be spread, it is important to address questions concerning synthetic textiles. Use of synthetic materials in textiles and clothing garments has increased from an annual production of 2.1 million tons of synthetic fibers in 1950 to almost 50 million tons in 2010 (Aizenshtein 2012; Essel and Ahrens 2015). Synthetic fibers account for approximately 60% of the total global fiber production, and polyamide (nylon) and polyester (polyethylene terephthalate (PET)) dominate. As textile production continues to increase worldwide, the total numbers of fibers entering into and passing through waste water treatment plants will inevitably increase.

Shedding, although well-known for anyone handling textiles, is a complicated phenomenon embracing a wealth of mechanisms, including both tensile fracture and flex fatigue of fibers as well as splitting or peeling off from the very fiber surface, in addition to the transport mechanisms away from the fabric (Hearle et al. 1998; Hearle and Morton 2008). Both loads acting normal to the fiber as well as frictional and shearing loads acting in angles less than 90°are anticipated during washing. Pilling (Doustaneh et al. 2013), a separate phenomenon on its own, also adds to the complexity.

Due to the importance of textiles as a source of microfibers in the environment, we have conducted a study aimed at quantifying fiber loss for several types of textiles. While the textile industry conducts research concerning design, techniques, and technological advances used in production of textiles, these studies do not address the implications of technological techniques and developments with regard to shedding of fibers, and as a source of microplastics. The aim of this work was therefore to address these issues and to test whether different knitting techniques, or use of different synthetic fibers, mitigate fiber loss. We also aimed to carefully quantify fiber loss with repeated washing and with wearing of fabrics. The information provided by this study could be of great importance to manufacturers, policy makers, and consumers looking for means by which to reduce release of microplastics or synthetic fibers into the environment, i.e., changes in practices used in textile production could potentially reduce microfiber release, and standardization of these practices could be implemented within industry.

Specifically, we hypothesized (H_1_) that fleece will shed more than other type of fabrics, that (H_2_) the knitting technique will impact where more tightly knit materials will shed less, and that (H_3_) different polymeric materials could behave differently. In addition, we aimed to test (H_4_) whether used/damaged clothes shed more fibers than new items. Since use of detergent in assessing the release of fibers during washing of garments has been in question (Browne et al. 2011a; Napper and Thompson 2016), we addressed (H_5_) the use of detergent in washing methods and whether this will influence the amount of fibers shed. Finally, the hypothesis (H_6_) was put forward that the number of washings has an impact.

## Materials and methods

### Fabrics

Here, we studied polymer fabrics consisting of either polyester (polyethylene terephthalate), the most dominant synthetic fiber on the textile market (Aizenshtein 2012; Carmichael 2015), polyacrylic (polyacrylonitrile), or polyamide (commonly known as nylon). Most of the fabrics were produced in the textile knitting laboratory at The Swedish School of Textiles. For the purpose of this work, we manufactured our own fabrics, thereby gaining full control of the treatment history of the fabric (including washing). When purchasing garments, this information and control concerning handling or treatment of fabrics is lost. These were produced using a Camber Velnit N.S. single machine no. 12210 size 26″ (for fabrics produced with E18 gauge) or a Monarch single machine model RX-SDY size 26″ (for fabrics produced with E28 gauge). However, some fabrics were commercial, provided by Tenson AB (Askim, Sweden); see Table 1 for details. After production, 10 × 10 cm pieces of cloth were cut using a laser cutter, GCC LaserPro Spirit GLS (settings; speed 9.4, power 93, PPI 1417). This was done in order to avoid loss of fibers from the cut edges of fabric, as the edges were melted. Fabrics were then dyed using a jet machine, Mathis Lab-Jet JFO (Mathis AG, Oberhasli, Switzerland), thereby making it possible for us to distinguish between fibers from different fabrics during subsequent analyses, and to identify any possible contamination. This was done according to the ISO standardized method for color fastness to domestic and commercial laundering; SS-EN ISO 105-C06. Some of the fabrics were repolished to mimic wear and tear, for example, that which occurs in clothing during use (see Table 1). This was performed using a Black & Decker KA85 Belt Sander 75 × 457 mm, with an abrasive belt cloth of 60 grains, for 1 s using a pressure of 5 kg. This method of wearing the fabric, resulting in visual damage similar to normal wear and tear, was developed in our lab.

**Table 1:** Description of textiles used in this study

	Polymer composition	Common name	Structure of fibers in textile	Abbreviation	Origin	Repolished/worn	Washed without detergent
**A**	Polyethylene terephthalate	Polyester	Knit (E18 100/36)	PET-1	SST	Yes	
**B**	Polyethylene terephthalate	Polyester	Knit (E18 100/144)	PET-2	SST	Yes	
**C**	Polyethylene terephthalate	Polyester	Knit (E28 100/36)	PET-3	SST	Yes	
**D**	Polyethylene terephthalate	Polyester	Knit (E28 100/144)	PET-4	SST	Yes	
**E**	Polyethylene terephthalate	Polyester	Knit, staple (E18 Nm 24/1)	PET-S	SST	Yes	
**F**	Polyacrylic	Acrylic	Knit, staple (E28 Nm 32/2)	A1	SST	Yes	Yes
**G**	Polyamide	Nylon	Knit (E28 44/13/2)	N1	SST	Yes	
**H**	Polyethylene terephthalate	Polyester	Microfleece	PET-PtMF	Polartech		Yes
**I**	Polyethylene terephthalate	Polyester	Microfleece	PET-TMF	Tenson		Yes
**J**	Polyethylene terephthalate	Polyester	Fleece	PET-TFL	Tenson		Yes

*PET* polyethylene terephthalate (polyester), *A* acrylic (polyacrylonitrile), *N* nylon (polyamide), *MF* microfleece, *FL* fleece, *Pt* Polar Tech, *T* Tenson. Supplier of commercial fabrics are shown. *SST* Swedish School of Textiles. The specifications describing the textiles indicate the knitting gauge, density, and number of filaments in the fibers. Size of each fabric was 10 × 10 cm, *n* = 6

The following terms are used to describe the structure of the fabrics. Yarns are the basic constituents of any fabric and come in two types, *filament* and *staple*. These two differ in length, where filament is continuous and staple yarns are short (typically order of mm to cm) and spun together. Originally, all manmade fibers are filaments. However, these filaments can be cut up into pieces to produce staple fibers. Yarns constructed from filaments can be identified using standard descriptions of the weight and number of filaments. Here, for example, 100/144 was used, giving the weight of 10,000 m in grams (100), and the number of filaments (144). Staple fibers typically have attractive comfort properties (moisture management, pliability, etc.). Both staples as well as multifilament yarns were used in this study. Fabrics can also be described as *knitwear*, a fabric kept together by yarns bound together in interlocking loops. Different *constructions*, i.e., patterns, could be used. In this study, single jersey is used. *Gauge* refers to the number of loop-forming needles per inch (E18 or E28), equivalent to the number of loops per distance, in turn measuring loop and fabric density. *Microfleece* is knitted terrycloth which is mechanically napped and/or cut. Napping is a textile process whereby fabrics are fed over revolving cylinders creating a soft, velvety appearance. See Table 1 for a list of tested fabrics.

### Washing of fabrics

All fabric samples were pre-washed in a Wascator FOM 71 Mp washing machine (Electrolux AB, Sweden) at 40 °C for 15 min, according to standards SS-EN ISO 6330, to remove loose fibers and dust but also to eliminate any differences between our manufactured fabrics and those we bought, since production processes will inevitably result in differing contamination of fabric with spin oils and other substances. No detergent was used during the pre-wash steps. The main washing steps used in production of experimental waste water for fiber analyses were conducted using a Gyrowash one bath 815/8 (James H. Head & Co Ltd., Halifax, England). This washer is a standardized laboratory washer, comparable to the Wascator but on a smaller scale, and is constructed to mimic a household washer, but with a closed container. Its use, as opposed to commercial washers, allows for further standardization of studies addressing textile shedding and washing procedures, as this research field grows. The Gyrowash uses steel cylinders with smooth surfaces and a volume of 255 mL. In order to mimic real household washing, 25 small stainless steel balls, 6 mm in diameter, are added. This is according to standard procedures and adds mechanical impact similar to the perforated and corrugated drum in machines like the Electrolux Wascator. Samples were washed according to SS-EN ISO 105-C06 standard protocol for color washing with one modification: sample sizes were 10 × 10 cm and not 10 × 4 cm. Samples were washed at 60° for 30 min, individually in separate container in the Gyrowash. Fabric was removed from the cylinder with tweezers and rinsed five times in 2 mL of water (10 mL in total), to simulate rinsing and to remove remaining loose fibers.

Fabric samples were washed with detergent. We used a commercially available detergent Liquid Via color (Unilever) as follows; 125 mL of water and 375 μL of liquid detergent were mixed and added to a Gyrowash steel container with 25 small metal balls. This detergent is known to contain C12–15 pareth-7, sodium laureth sulfate, potassium cocoate, and TEA-Cocoate. Several of the fabrics were also washed without detergent since a previous study (Browne et al. 2011a), only reported results from garments washed with water alone, see Table 1.

### Filtering of water

All laboratory materials used in this experiment were thoroughly rinsed to reduce risk of contamination, as were work stations. Wash water and rinse water from each fabric (*n* = 6 for each type of textile/treatment) were collected in glass beakers. Prior to filtering the water, the glass container was lightly shaken to remove fibers adhered to the sides of the bottle. A glass filter (Whatman GF/C, pore size 1.2 μm) with a pore size of 1.2 μm, diameter 42.5 mm, was placed in a vacuum filtration assembly with glass collection flask, attached to a vacuum pump. Water from one sample at the time was poured carefully through the filter, and samples flasks were rinsed with ultrapure milliQ water to assure that no fibers were lost on the sides. The filter was moved to a thoroughly rinsed glass petri dish with cover using a pincette, prior to microscopic analysis.

### Microscopy

The glass-fiber filters were divided into four areas, and each of these areas was divided further into another four areas (total 16 areas on each filter). This was done to allow for easier and accurate manual counting of the fibers. Filters were analyzed using a Carl Zeiss: 475,002–9902 Lyca light microscope with a magnification of 40×. Fibers in each area were counted; the entire filter was assessed for samples with few fibers but only half of each filter was quantified in cases where samples released large numbers of fibers. For samples with a low number of fibers, all fibers were further classified and quantified by size on a scale from 1 to 5 where 1 was smallest and 5 was longest. In samples with a large amount of fibers, representative fibers were measured and classified within a limited number of areas on the filter. Size 1 = 0.025 mm–0.25 mm, size 2 = 0.25 mm–1 mm, size 3 = 1 mm–1.75 mm, size 4 = 1.75 mm–3 mm, and size 5 = > 3 mm.

### Statistics

In all cases, six fabric swatch replicates were used for each test (*n* = 6), unless otherwise indicated. Results showed unequal variance indicating the need for data transformation. T tests were used to compare results between two fabrics or treatments. ANOVA with repeated measures was used to test the effect of fiber loss during sequential washes. Group wise comparisons were conducted using ANOVA tests, *α* = 0.05. Student-Newman-Keuls post hoc analyses were applied. All data was subjected to Levine’s test of homogeneity of variances and normal distributions. All data was analyzed using SPSS 23.

## Results and discussion

**Fig. 1:**
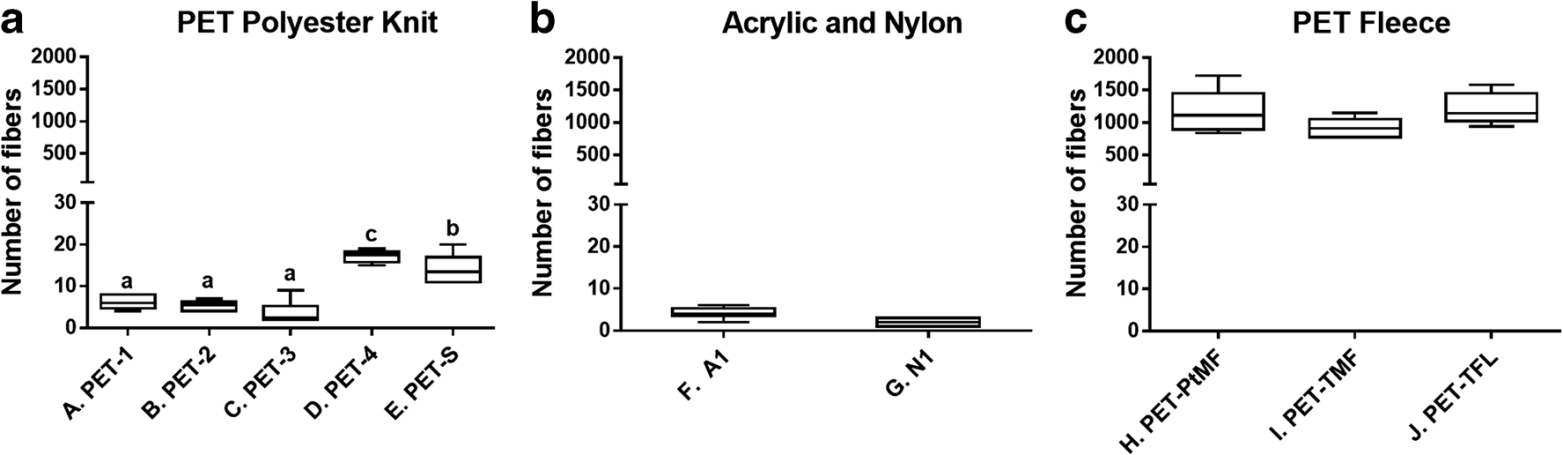
Total number of fibers released from 100 cm^2^ of fabric per wash. Results are presented in box plots showing median, 25th, and 75th percentiles, max and min. Different types of fabrics are divided into separate figures; note that *y*-axis scale is identical in all figures, to emphasize differences. **a** Five polyester (PET) fabrics of differing structure. **b** Acrylic (A1) and nylon (N1). **c** Three different polyester (PET) fleece (FL) or microfleece (MF) fabrics, commercially produced by Polar Tech (Pt) or Tenson (T). Statistically significant differences are indicated by letters (*p* < 0.05). See Table 1 for more detailed descriptions of fabrics. *n* = 6

The results from our analyses indicated that, as we predicted, fleece fabrics do release a significantly greater number of fibers when washed, compared to the other knit fabrics (*F* = 72.982, 58; *p* < 0.001), allowing us to reject our first null hypothesis. These results are depicted in Fig. 1. The knit fabrics, averaged together, released 9 ± 7 fibers per 100 cm^2^ of fabric. Fleece and microfleece (fabrics H–J, see Table 1) did in fact release an average of 1177 ± 135, 932 ± 59, and 1210 ± 96 fibers, respectively, per 100 cm^2^ of fabric. This is a much greater amount than the 1900 fibers per garment previously reported (Browne et al. 2011a), but perhaps closer to levels more recently reported (700,000 fibers per 6 kg wash) (Napper and Thompson 2016). Typically, around 1m^2^ of fabric is used for a garment for adults giving a scaling factor of 100 for comparison between our 100 cm^2^ sample and an average garment, indicating that one fleece garment could release approximately 110,000 fibers. These values are however difficult to compare to previous studies since the authors did not report fabric area, weight, or water volume in all cases. The seven other fabrics tested here also released fibers, though at lower amounts, with an average of nine fibers per test or approximately 900 fibers per garment.

A comparison of the knit polyester fabrics knit with different techniques (labeled A-E, see Table 1) revealed significant differences (*F* = 48.044, 41; *p* < 0.001); our second null hypothesis can therefore be rejected. Post hoc testing revealed that two types of polyester knits tested, tightly knit polyester and a knit staple, released more fibers than the remaining three polyester fabrics. The following relationship between fabrics in terms of fibers shed: PET-4 > PET-S > PET-1, PET-2, PET-3. One polyester fabric (PET-4, E28 100/144) released twice as many fibers as three of the other fabrics knit from filament yarn (PET 1–3, or A–C in Table 1), indicating that materials knit from yarns with a greater number of exposed filaments per area shed more fibers compared to fabrics made with yarns with fewer filaments. Here, the needle gauge and the yarn are important factors contributing to the release of fibers. The degree of shedding is related to how tightly the yarn is knitted into the fabric. More tightly knitted fabric, as indicated by the knitting gauge (for example, DE28), results in more fibers on the same area of fabric resulting in a greater fiber loss. See Fig. 1a and Table 1. Sample E (PET staples, PET-S) was also found to have a larger degree of shedding than the first three polyester fabrics tested (A–C) indicating that the staple yarns may also be a less desirable structure, from an environmental perspective. This has implications for textile industries where efforts are being made to produce fabrics that shed less and are therefore more environmentally friendly. Our tests did not find any significant differences between the three different fleece fabrics tested here (*F* = 2.478, 17; *p* = 0.165), nor did we find a difference between nylon and acrylic fabrics. Nylon and acrylic fabrics were found to shed fibers in similar amounts to PET 1–3 (*F* = 1.848, 41; *p* = 0.082). See Fig. 1b, c.

**Fig. 2:**
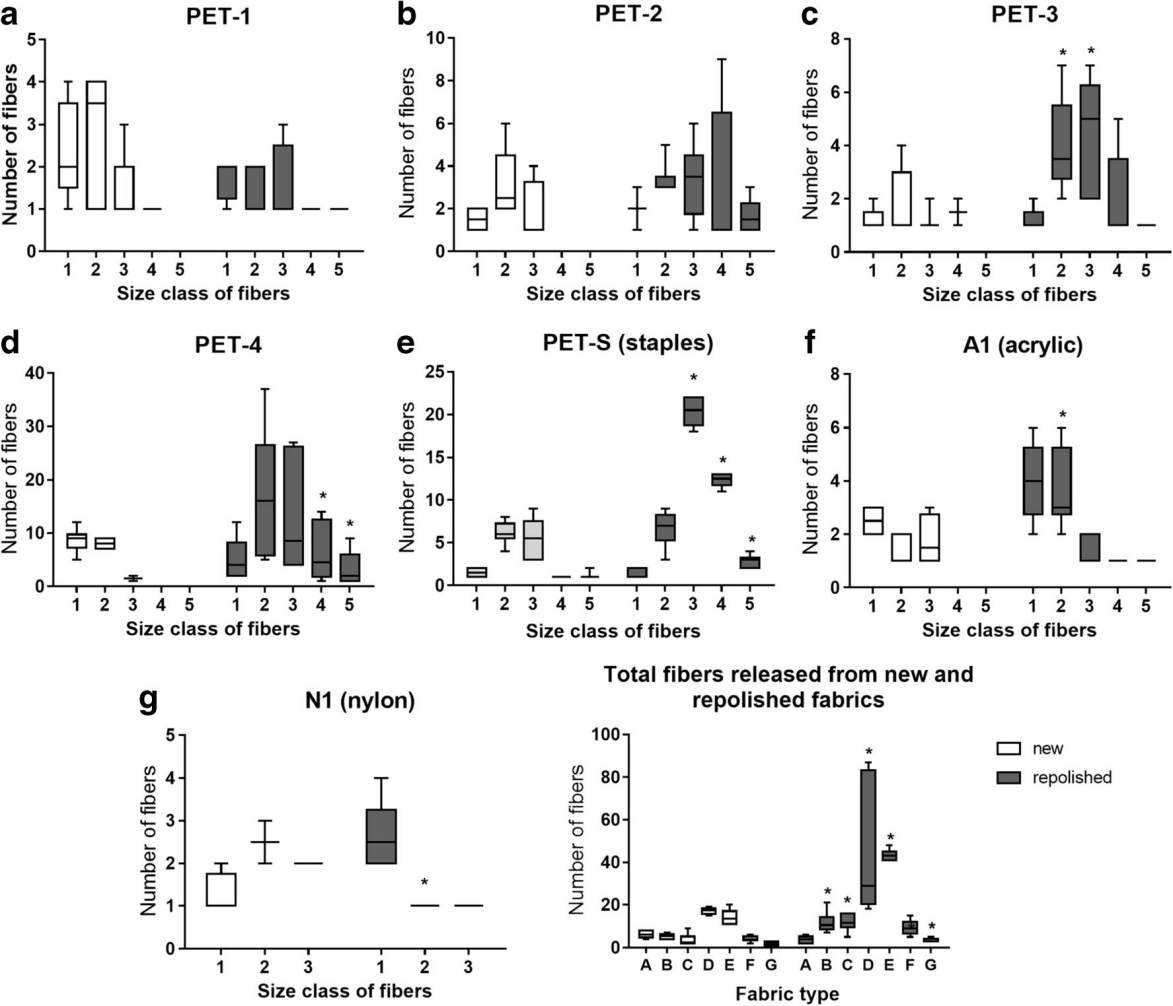
Total number of fibers released from 100 cm^2^ of fabric per wash, using new and repolished (worn) fabrics: five polyester (PET) fabrics of differing structure, acrylic (A1) and nylon (N1). Results are presented in box plots showing median, 25th, and 75th percentiles, max and min. Results are presented together in final graph for easier comparison; letters indicating fabric type correspond to the previous graphs in this figure and to Table 1. Size 1 = 0.025 mm–0.25 mm, size 2 = 0.25 mm–1 mm, size 3 = 1 mm–1.75 mm, size 4 = 1.75 mm–3 mm, size 5 = >3 mm. Statistically significant differences between washes for each fabric are indicated by (*p < 0.05); *n* = 12; *y* axes differ in scale

The number of microplastic fibers released during washing could be impacted by aging of garments, i.e., through usage and passage of time. A recently published report, commissioned by Patagonia (producers of outdoor clothing) found that aged garments indeed shed higher masses of fibers than newer garments (Bruce et al. 2016), and we had hypothesized that this would be the case in the current study as well. Several fabrics were repolished to mimic wear and tear, and our results (in Fig. 2) indicate that repolishing fabrics generally resulted in greater fiber loss compared to non-repolished, i.e., new, fabrics. The total number of fibers released from the repolished fabrics was significantly larger for all tested materials with the exception of PET-1. These results are shown in Fig. 2. The *p* values obtained from *t* tests for four different PET knits (PET 1–4), nylon, and two fleece fabrics (PET-PtMF and PET-TMF) were as follows: 0.061, 0.013, 0.002, 0.041, 0.007, 0.013, and < 0.001, respectively. We also classified and quantified fibers of different size (categorized as class 1–5). We found that for polyester knits (PET 2–4) and PET staples, repolishing resulted in an increase in the release of longer fibers while the opposite was true for nylon and acrylic.

**Fig. 3:**
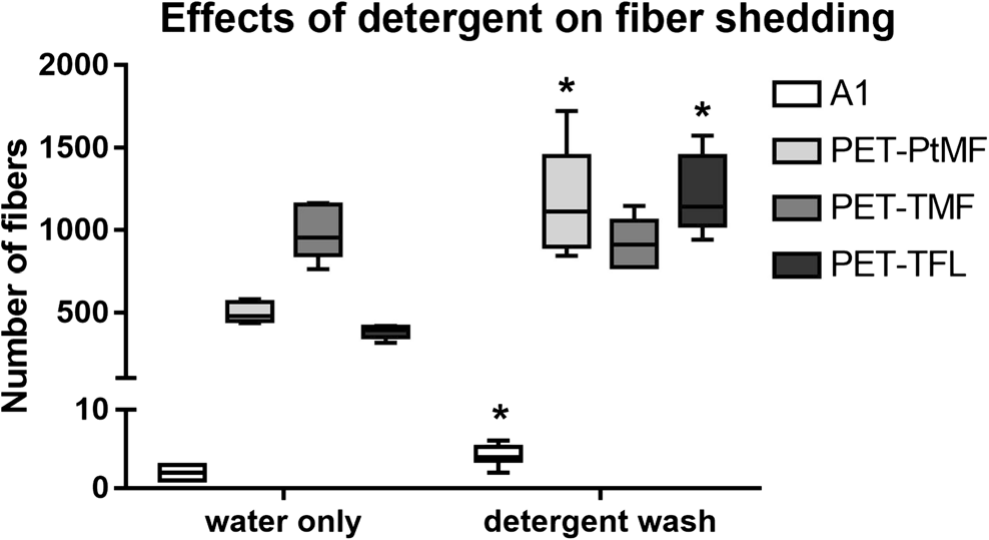
Total number of fibers released from 100 cm^2^ of fabric per wash, when fabrics were washed with water alone or with a commercially available detergent. Fabrics tested: acrylic (A1), and polyester (PET) fleece (FL) or microfleece (MF) fabrics, commercially produced by Polar Tech (Pt) or Tenson (T). Results are presented in box plots showing median, 25th and 75th percentiles, max and min. Statistically significant differences between washes (with or without detergent) for each fabric are indicated by *, (p < 0.05). *n* = 6

It is important to note that our results are obtained from fabrics washed using a commercially available detergent, a factor that we included and tested in our fifth null hypothesis. We conducted a comparison of the number of fibers shed from a selected fabrics washed with and without detergent. Our data (Fig. 3) indicate that washing with detergent results in a significant increase in amount of fibers released for three out of four fabrics tested; *p* values obtained from *t* tests were 0.011, < 0.001, 0.560, and < 0.001 for acrylic (A1), Tenson fleece (PET-TFL), Tenson microfleece (PET-TMF), and Polartech microfleece (PET-PtMF), respectively. In the past, estimates of fibers shed from textiles were done with water alone, as effluent water containing detergent proved difficult to analyze since filters would tend to clog (Browne et al. 2011a). In the current study, we improved rinsing and filtering methods, allowing for a more realistic assessment of effects of washing routines as well as a better comparison of our results with previous work (Browne et al. 2011a). A more recent study has also shown that more fibers tended to be released from fabrics washed with bio-detergent and conditioner (Napper and Thompson 2016). These increases in fiber loss with the use of detergent are likely dependent upon a fundamental property of detergents; they decrease the surface tension. Knitwear, consisting of woven loops, forms a fabric surface that acts like a cage which incorporates particles including loose fibers, but the low surface tension resulting from detergent use is essentially equivalent to “better” wetting which in turn results in improved rinsing of the fabric, and whatever particles that are held in the 3D knitwear network might be released. In addition to this wetting aspect, another fundamental property of detergents is that they act as dispersing agents; dirt is dissolved in the medium (water) and kept there, thereby prevented from falling back onto the fabric. This property applies to fiber fragments as well; most fibers that are released from the fabric are also transported away and have an increased chance of being detected in the subsequent analysis. Our results, in concurrence with a recent study (Napper and Thompson 2016), indicate that levels of fibers released during washing are much higher than previously estimated.

**Fig. 4:**
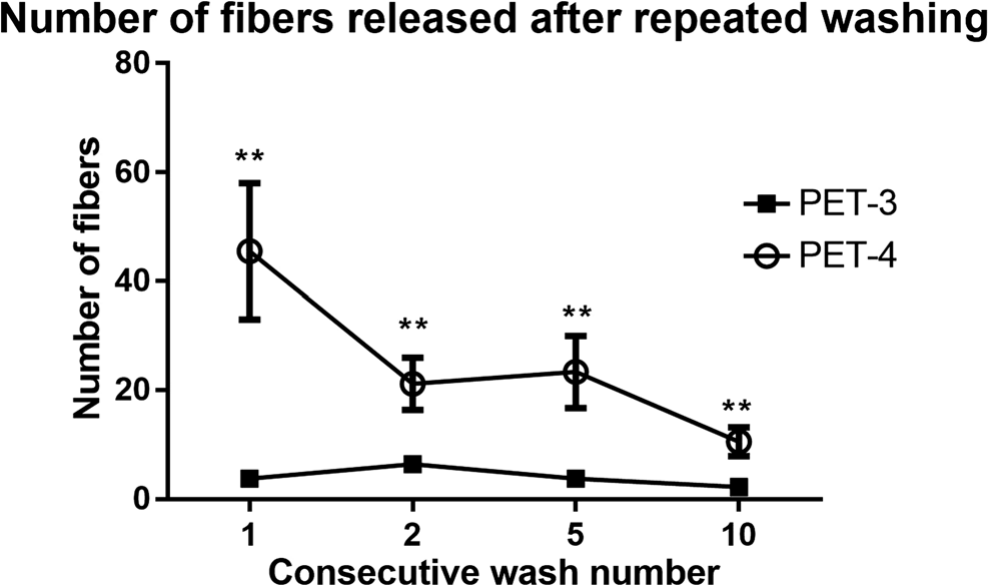
Number of fibers release after repeated washing from polyester fabrics knit with different gauges (PET-3, knit E28 100/36 and PET-4, knit E28 100/144). Results are presented as mean ± SEM. Statistically significant differences between number of fibers released from each fabric following the indicated number of washes are indicated by (**p < 0.01). *n* = 6

In order to further increase the accuracy and realism of our estimates of fiber release from PET fabrics, we repeatedly washed two of the fabrics, PET-3 and PET-4, and quantified the number of fibers released after two, five, and ten washes. These two fabrics were chosen for this experiment since they are among the most common types found in clothing, and showed a high degree of variation in number and sizes of fibers released in previous tests. Results, shown in Fig. 4, indicated that PET-4 released significantly greater numbers of fibers during each wash, compared to PET-3 (*p* < 0.01 for all four comparisons). The number of fibers released from PET-4 decreases significantly after repeated washes (*F* = 5.159, 3; *p* = 0.012), indicating that older clothing may release fewer fibers than new garments. This is also in concurrence with the results presented in recent studies (Napper and Thompson 2016; Pirc et al. 2016). However, since wear and tear increases fiber release, it is likely that fabrics will continue shedding as they age. We did not, unfortunately, test both of these parameters simultaneously.

The results presented in this study provide a strong indication that the shedding of fibers from clothing during washing is an important emitter of microplastics; our results, together with newly reported levels of shedding from fabrics (Napper and Thompson 2016; Pirc et al. 2016), confirm that previously reported levels were underestimated (Browne et al. 2011a). Downstream consequences of this have been addressed, but are not fully understood. Microplastic fibers are known to reach both aquatic and terrestrial environments via sewage; previous studies have shown that a large portion of plastic fibers may be removed during sewage treatment, from waste water to the sludge. Magnusson and coworkers reported 10.7 ± 0.39 and 4.00 ± 0.58 in 1 m^3^ incoming and effluent water, respectively (Magnusson et al. 2016; Magnusson and Wahlberg 2014). These studies have analyzed samples filtered through 300 μm filters (Magnusson and Wahlberg 2014), which could potentially lead to loss of small fibers and an underestimation of fiber levels in water.

While reported levels are substantial, estimating the relative importance of sewage effluent as a source of microplastics is difficult to estimate. There are a number of reports indicating the importance of urban areas on synthetic fibers in the environment (Browne et al. 2011a, b; Thompson et al. 2004), but we still lack knowledge about spatial and temporal distribution of microplastics, as well as accurate descriptions of particle compositions and breakdown in the environment. Nevertheless, there is enough evidence supporting a move toward caution. Reports on levels and effects of microplastics in the aquatic environment indicate real and potential threats (Galloway and Lewis 2016; Wright et al. 2013), but these are not limited to the water. Terrestrial environments also face threats; the accumulation of fibers in sludge is a possible risk factor; as microplastic fibers may spread via sewage (Habib et al. 1998; Zubris and Richards 2005) thereby contaminating land areas and impacting organisms (Huerta Lwanga et al. 2016; Palmqvist et al. 2016). Potential impacts on human health have also been discussed (Bouwmeester et al. 2015; Koch and Calafat 2009; Thompson et al. 2009).

## Conclusions

Our current knowledge concerning microplastics in the environment, together with the knowledge concerning fiber shedding presented here, provides a starting point for increased efforts to reduce environmental contamination by microplastics. We find several potential points of release of fibers from fabrics, i.e., during production, which could be improved using straight forward strategies. This begins with the first steps of textile production where companies may be inspired to choose or develop knitting techniques that reduce fiber loss. Furthermore, yarn choice has an impact, with tightly constructed yarns as the preferred choice. While the decrease in release of fibers after the first wash was not significant for all materials studied here, it could still be interesting to encourage industries to wash textiles prior to clothing production, thereby potentially decreasing the amounts of fibers released into the environment during the consumer phase. (Collected fibers would of course need to be handled properly to avoid release into the environment). Washing and rinsing and subsequent drying are common processes within the textile industry, and since this infrastructure is already in place, the cost for the consumer would be highly moderate. The same goes for another suggestion, namely adding an in-line vacuum system through which the fabric could be passed subsequent to any of the brushing, sanding, raising processes that are common in the textile industry for getting a better hand and comfort, thereby eliminating loose fibers via air filtration and exhaustion. This could potentially be done quickly, at full production speed. There is currently no industry standardized testing or labeling of garments which provides information concerning fiber release from fabrics, and we suggest that this could be explored further.

Reduction of fiber loss once garments reach consumers could be addressed via appliance manufacturers of washers and dryers who are encouraged to develop means of preventing fiber loss in wash water effluent, as has been previously suggested (Browne 2015). Further, downstream, the installation of additional filters for outgoing sewage effluent could prevent these fibers from reaching the environment. Finally, individual consumers are encouraged to purchase fabrics that release fewer fibers, and perhaps to wash them less often; airing clothing in sunlight after use will often reduce the number of washes for certain types of garments (i.e., outerwear).
